# Microwave Absorbing properties of metal functionalized-CNT-polymer composite for stealth applications

**DOI:** 10.1038/s41598-020-72928-1

**Published:** 2020-09-29

**Authors:** Mousa I. Hussein, Syed S. Jehangir, I. J. Rajmohan, Y. Haik, Tahir Abdulrehman, Q. Clément, N. Vukadinovic

**Affiliations:** 1 United Arab Emirates University (UAEU), Al Ain, 15551 UAE; 2 Texas A & M University-Kingsville, Kingsville, TX 78363 USA; 3 Hamad Bin Khalifa University, Doha, Qatar; 4 Dassault Aviation, 92552 Saint-Cloud, France

**Keywords:** Materials science, Nanoscience and technology

## Abstract

In this study, we report on the electrical properties of multi-wall carbon nanotubes (MWCNT) composites functionalized with metal or metal alloy oxides and embedded in a polyurethane matrix to develop a lightweight material for microwave absorption and shielding. The CNT nanoparticles are functionalized with metallic oxides such as Cobalt oxide, Iron oxide, and Cobalt Iron oxide, at three different concentrations. Metallic oxides are used at 5%, 10%, and 20% concentration of the total CNT percentage weight. The resulting functionalized CNT is mixed with polyurethane polymer at 5% wt of the total composite weight. Three sets of cylindrical samples are developed, and each set contains three different metal oxide concentrations. The dielectric properties of the nine developed samples are obtained by measuring their permittivity spectra using an open-ended coaxial probe technique in the spectral range 5–50 GHz. The absorption efficiency of the composites is then obtained by calculating the reflection loss at normal incidence. The results show that the spectral range of absorption can be tuned by changing the CNT concentration, and the material thickness. Functionalized CNT with different alloyed metal oxides enhanced the absorption efficiency of the polyurethane/CNT composites. Such functionalized composites can be used to replace the common heavyweight materials used for microwave applications.

## Introduction

The rapid advancement and widespread of microwave and RF communication systems over the years, has led to an abundant increase in electromagnetic energy radiation in our living environment. Such an increase in microwave sources is due to the development and advancement in communication techniques (mobile phones, laptops, antennas for aeronautics, or automobile) and of the electronic warfare in the military field (radar, Satellite). Recently research efforts are focused on finding solutions to guarantee protection from electromagnetic (EM) radiations. Different types of composite materials have been developed in order to overawe this issue, for example, to reduce the electromagnetic interferences with communication devices, or can be deposited onto wind turbines to reduce their interference with radar signals (weather, air traffic control, sky monitoring). Nonetheless, absorbing material has significant importance in military aircraft for stealth applications, for which they must be as light as possible to avoid weight overload.

Advancement in material science and engineering has led to the development of composite materials with great microwave absorption capabilities while taking into account the weight constraint, which is an essential factor in aeronautic applications. This kind of material is developed by impeding absorbing charges (magnetic or dielectric) into a host matrix material. Magnetic charges based on ferromagnetic metals such as iron, nickel, cobalt or their alloys are often used as they enable to have a strong absorption in several spectral ranges of interest, due to both dielectric and magnetic losses^1^. However, composite materials, including magnetic charges, are usually heavy and thus lead to a substantial overweight. On the other hand, carbonaceous particles are proven to be excellent candidates for designing high-performance microwave absorbers^2^. Carbon comes in various allotropic forms, among which the carbon nanotubes that correspond to a hexagonal mesh formed in cylindrical nanostructure^3^ have attracted scientific community and researchers' attention. CNT comes in either single-wall CNT (SWCNT) or multi-wall CNT (MWCNT). The stacking of the CNT cylinders in a proper polymer results in a lightweight and potentially broadband microwave absorbing materials, while keeping good thermal and mechanical properties^4,5^. In addition, functionalizing the CNT by filling^6–10^ or coating^11–13^ with magnetic nanoparticles allows tuning the microwave absorption properties. However, the vast majority of the published results deal with composites where the CNTs are dispersed in an epoxy resin matrix, or to a lesser extent, in polyethylene, wax, or paraffin host media. In the context of aeronautics applications, polyurethane matrix, which has been already certified, is usually preferred due to its excellent mechanical properties (flexibility, resistance to bending, good properties in a wide temperature range) and easiness to be produced in great quantity (panels of several m^2^), but scarce investigations have been conducted on polyurethane/CNT composites as microwave absorbers^14^. The interaction of the electromagnetic energy with the material is highly related to the electrical properties of the material. Such interaction is related to material composition and content that can interact with the electric and magnetic forces produced by the stimulus electromagnetic fields. From an energy point of view, this interaction can be translated as energy storage, which describes energy dissipation (absorbed) inside the material; and energy storage, which relates to the lossless portion of energy exchange between the stimulating field and the material.

This work presents the synthesis process used to produce MWCNT, then a comparison of the dielectric properties between basic and functionalized CNT composites embedded in polyurethane (PU) will be discussed for the spectral range 5–50 GHz. The polyurethane (PU) matrix was mixed with 5 wt% functionalized CNT. In this study the 5 wt% CNT was functionalized with 5%, 10%, and 20% of Cobalt oxide (Co oxide), Iron oxide (Fe oxide), and Cobalt iron oxide (CoFe oxide). Results for the real and imaginary parts of the dielectric constant along with the loss tangent are presented for frequency range from 5 to 50 GHz. The absorption efficiency of the CNT composites will be studied by calculating the reflection loss for the measured samples with different sample thicknesses.

## Materials and methods

### Basic CNT production

The synthesis of the MWCNT has been carried out using the chemical vapor deposition (CVD) technique. The developed system enables the production of MWCNT, with a yield of few grams per hour, by inserting pure gas containing carbon, nitrogen and hydrogen into a quartz tube heated in an oven at a temperature of 720 °C. Metal nanoparticles such as cobalt or nickel are used as catalysts in the quartz tube to initiate the chemical reaction that allows the growth of the CNT. After the synthesis, the CNT need to be purified. The purification is done by using a patented ionic liquid based technology, developed at the United Arab Emirates University, and leads to the production of 95% pure MWCNT. The produced CNT is of inner diameter between 5–12 nm, outer diameter is 30–50 nm and the length is 10–20 µm.

### Functionalized CNT

Functionalization consists of adding magnetic nanoparticles inside or outside the CNT. This process is usually done with a low content of nanoparticles. This condition is imposed such that the composite does not have a significant magnetic loss. The magnetic nanoparticles can be inserted inside the CNT (filled CNT) or adsorb on their external surface (coated CNT). The first solution usually leads to better dielectric properties but is more expensive and more difficult to develop. Therefore, we carry out the CNT functionalization with the second solution.

Synthesis of nanoparticles: The Cobalt iron oxide (CoFe oxide [CoFe_2_O_4_]) nanoparticles were prepared by the co-precipitation reaction. 0.1 M of Cobalt nitrate solution and 0.1 M of Ferric nitrate solution were mixed in the appropriate ratio and made up to 200 mL with deionized water. For single metal nanoparticles, either cobalt nitrate or ferric nitrate was used for the reaction. The solution was heated up to 90^0^C with continuous stirring using a teflon rod. To this solution 8 M sodium hydroxide solution was added dropwise resulting in the formation of a brownish-black solution. The mixture was heated at 90^0^C with stirring for 90 min. The nanoparticles were separated by magnetic separation, followed by washing several times to remove impurities. The nanoparticles were finally dried at 50 °C.

The CNT functionalization has been done with (Cobalt oxide, iron oxide, and cobalt-doped iron oxide nanoparticles). Metal oxide nanoparticles are sonicated in 100 mL of distilled water for 5 min in an ice bath. The CNTs are also sonicated separately in 100 mL of distilled water for 5 min in an ice bath. Then, the CNT solution is poured into the nanoparticles solution with magnetic stirring for 12 h. The CNT functionalization is then obtained by adsorption of the nanoparticles onto the external surface of the CNT. A change in the color of the supernatant indicates that the adsorption phenomenon has happened. The functionalized CNTs are finally collected by centrifugation at 3000 rpm and are then dried in order to be used for crafting composite samples.

Preparation of Polyurethane-CNT: The nanoparticle [Cobalt oxide (Co oxide) or Iron oxide (Fe oxide [Fe_3_O_4_]) or Cobalt iron oxide (CoFe oxide)] functionalized carbon nanotube (CNT) in Polyurethane was prepared by adding the required amount of functionalized CNT (5 wt%) in 70 g of Reckli-FM-PU 48 polyurethane base (Polyol mixture) and was heated to 55 °C followed by thorough mixing. The Polyurethane-CNT mixture was then sonicated for 15 min to ensure uniform distribution of the CNTs in the polyurethane base. After sonication, the polyurethane base with CNT was allowed to cool to room temperature followed by the addition of 7 g of Reckli-FM-PU 48 hardener. The Polyurethane-CNT and hardener mixture were mixed thoroughly and poured into a mold release agent coated hollow metal cylinder closed at one end. The Polyurethane-CNT-hardener mixture was dried at room temperature for 24 h and removed from the metal cylinder to obtain solid PU-CNT composites with a diameter of 1 inch and a height of 4 inches.

**Figure 1:**
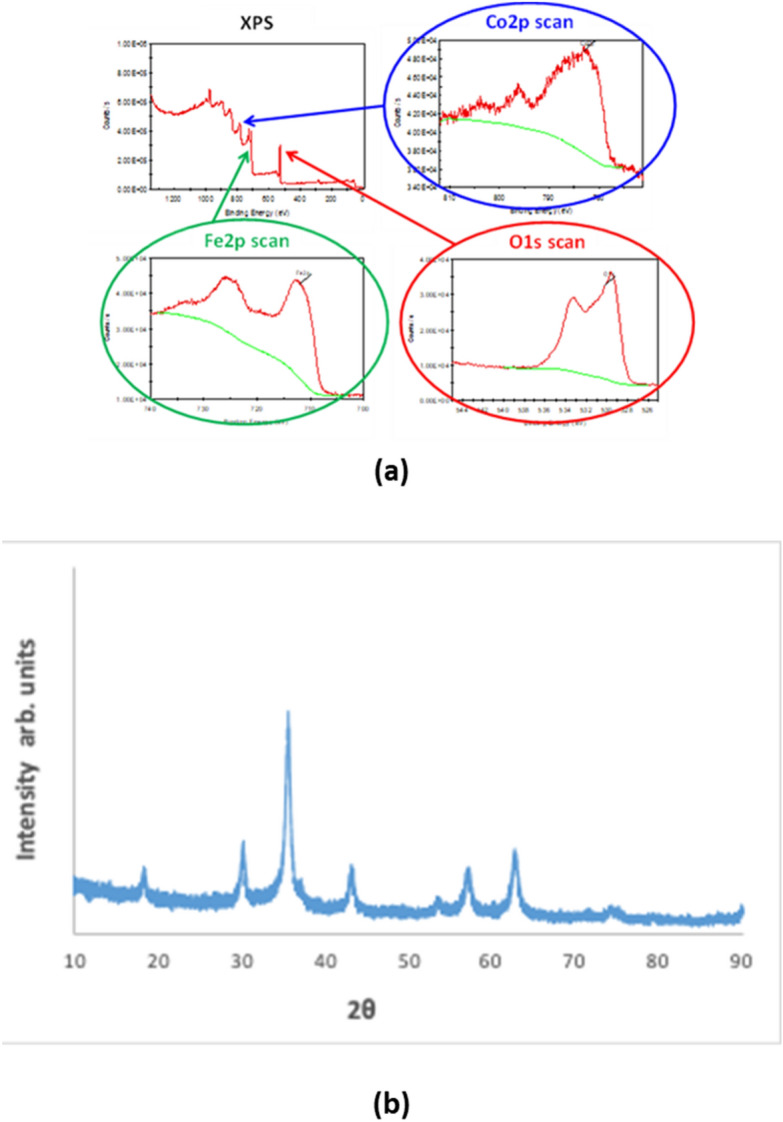
(**a**) XPS spectra of cobalt iron oxide nanoparticles. (**b**) XRD of the Cobalt iron oxide nanoparticles.

**Figure 2:**
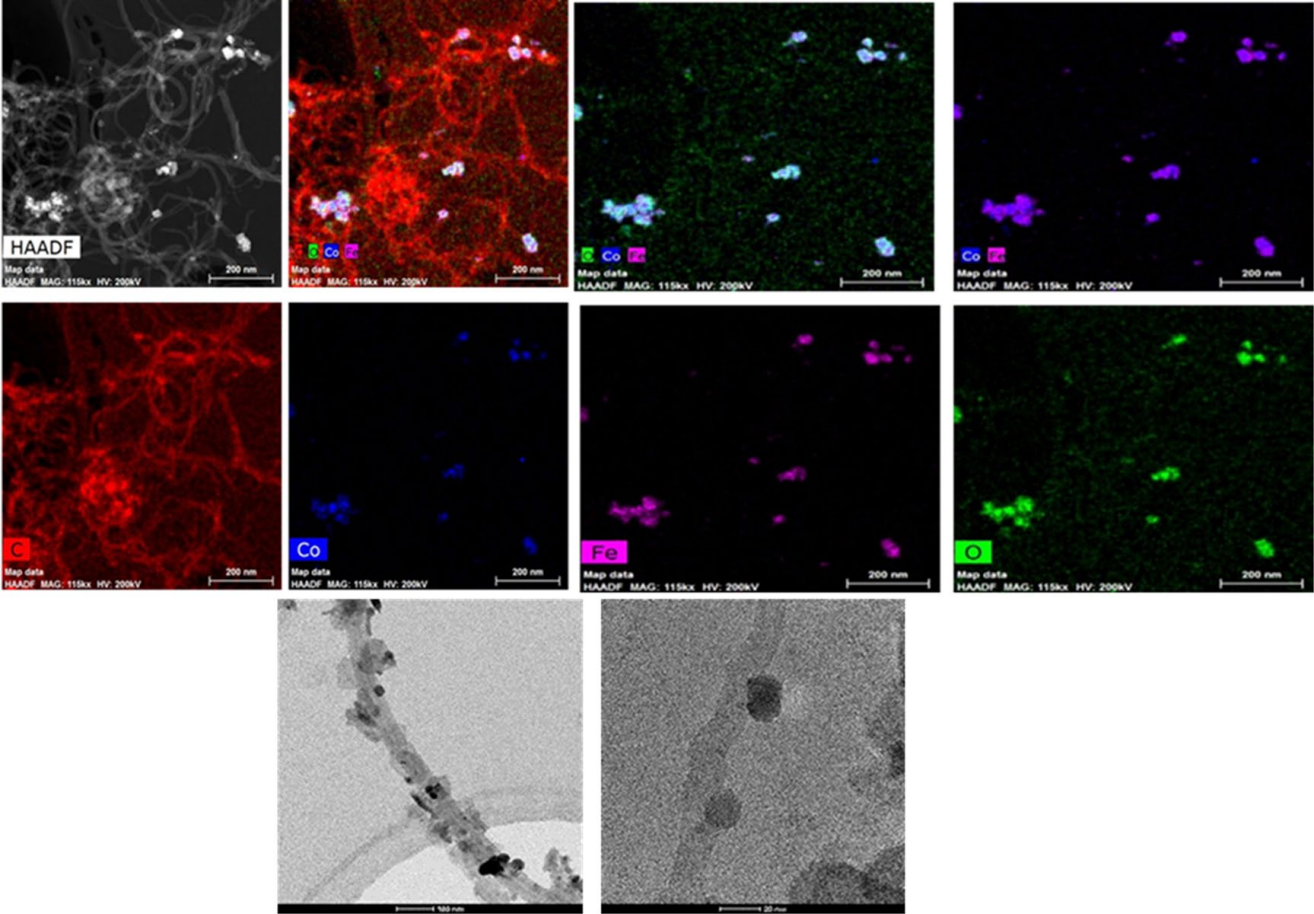
The upper two rows show the TEM imaging of the elemental mapping of Cobalt iron oxide nanoparticles, the third row show the TEM images for CNT Cobalt-iron oxide particles.

**Figure 3:**
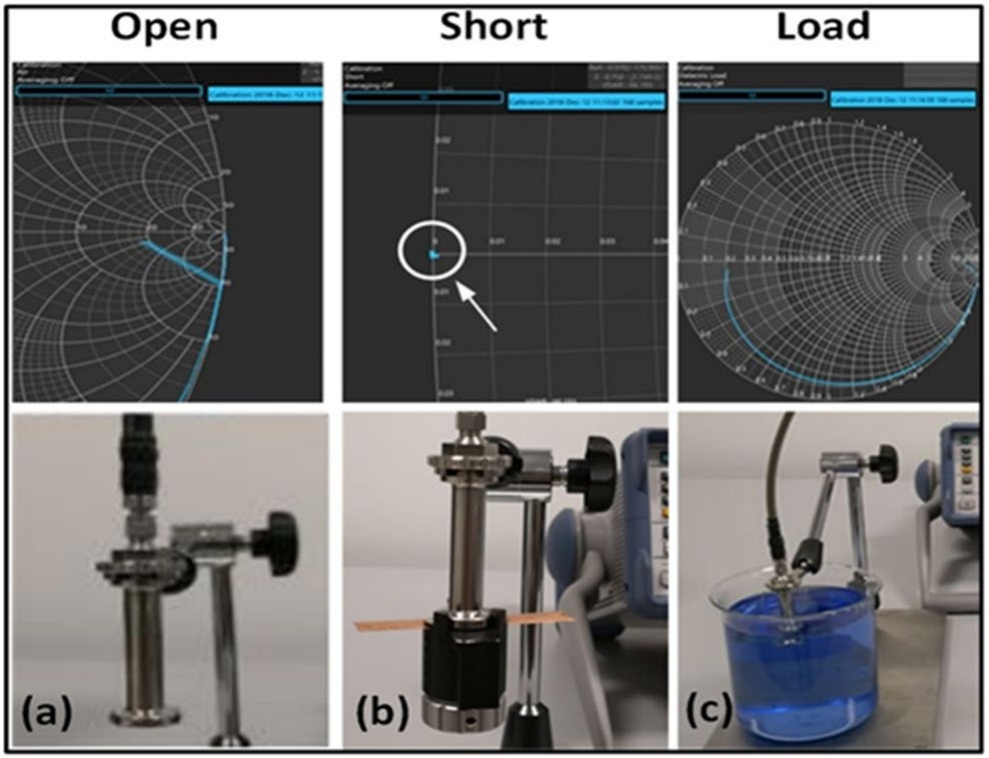
OSL calibration setup for the measurement.

The XPS data reveals the presence of cobalt iron oxides sample and are determined using Escalab 250 Xi (Thermo Fisher Scientific). The XPS spectra of O1s, Fe2p and Co2p along with the survey scan of cobalt iron oxide are presented in Fig. 1a. The atomic percentages of cobalt, iron and oxygen determined by XPS were found to be 10.8%, 31.2%, and 58% respectively. The TEM image for the elemental mapping of Cobalt iron oxide are presented in Fig. 2. The CO and Fe are mapped individually and in combination in the upper two rows of Fig. 3. The lower two images in Fig. 3 shows the morphology of the nanoparticles attached to CNT. The morphology indicates spherical like particles. The XRD (Fig. 1b) of the cobalt iron oxide nanoparticles was determined using Bruker D8 advance instrument. The XRD diffraction pattern of cobalt iron oxide nanoparticles can be compared to that of CoFe2O4 and corresponds to the JCPDS data (No. 221086) given by Mitra et al.^15^ where the peaks were indexed to FCC structure of CoFe2O4. The diffraction peaks of the synthesized cobalt iron oxide nanoparticles indicate the crystallinity of the material. The XPS, XRD and TEM all confirm the formation of crystalline cobalt iron oxide nanoparticles.

## Microwave properties

### Permittivity measurement

Various experimental microwave techniques have been reported to obtain the permittivity and conductivity spectra of CNT-based nanocomposites, namely, stripline cavity^17^, coaxial line^7,10,12,14,16,18,20,24^, and a waveguide^5^. In our study, we chose to use the open-ended coaxial probe for its ease of calibration, wide frequency band coverage, and no special processes are needed in preparing the samples. However, this measurement technique will give the complex dielectric constant only^21^. It has been reported by several researchers, that the addition of magnetic Nano particles, such as Co and Fe will not significantly affect the complex magnetic permeability^22–25^.

In this study, the permittivity measurement was conducted using the open-ended coaxial probe^16,19,21,26,27^ covering frequency range 5–50 GHz. The Scattering parameters S_11_ was measured with a probe connected to Anritsu Vector Network Analyzer (VNA). The open-ended coaxial probe (DAK-1.2 Dielectric Probe) developed by SPEAG was used to measure and extract the dielectric properties of the samples. Then, DAK software was employed to derive the complex permittivity from the S_11_ measurement. System calibration is an important step for precise measurements. SPEAG recommends using three steps calibration process these are OSL, for open, short and dielectric load. The open calibration was established by exposing the probe to air. The short calibration was done by using a shorting block and metallic strip. Moreover, the load calibration was accomplished using de-ionized water, for which the dielectric properties are known as a function of frequency and temperature.

Calibration normalizes the magnitude and phase changes of the probe and hence normalizes the reflection coefficient measured by the network analyzer, this process moves the measurement reference planes to the end of the probe. The probe and the calibration setup are depicted in Fig. 3. Then, the measurement is carried out on the CNT composites, by placing the probe flange on the cylindrical samples of 1″ diameter and 4″ of length. For a reliable measurement, air gaps between the samples and the probe must be avoided.

**Figure 4:**
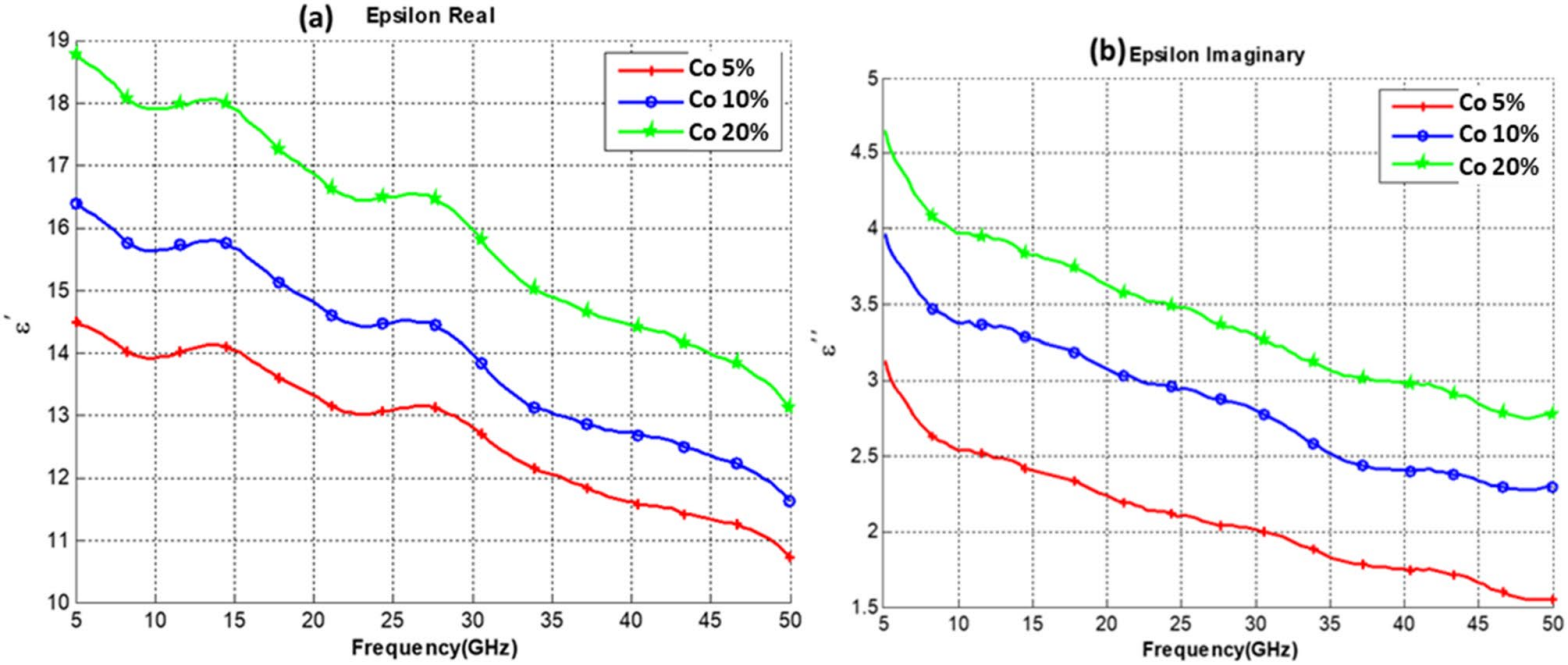
Dielectric Constant of PU at 5% CNT composites with different (Co) content. (**a**) Real Part. (**b**) Imaginary part.

**Figure 5:**
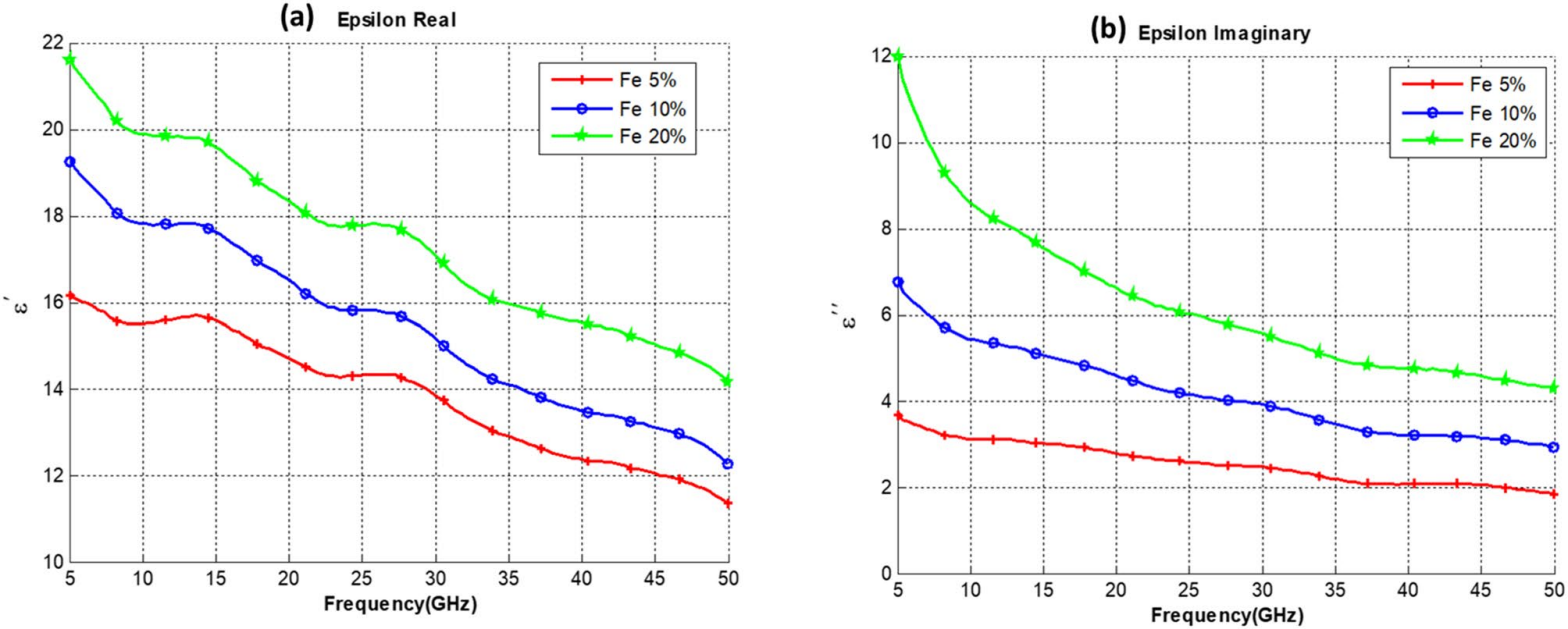
Dielectric Constant of PU and 5% CNT composites with different (Fe) content. (**a**) Real Part. (**b**) Imaginary Part.

**Figure 6:**
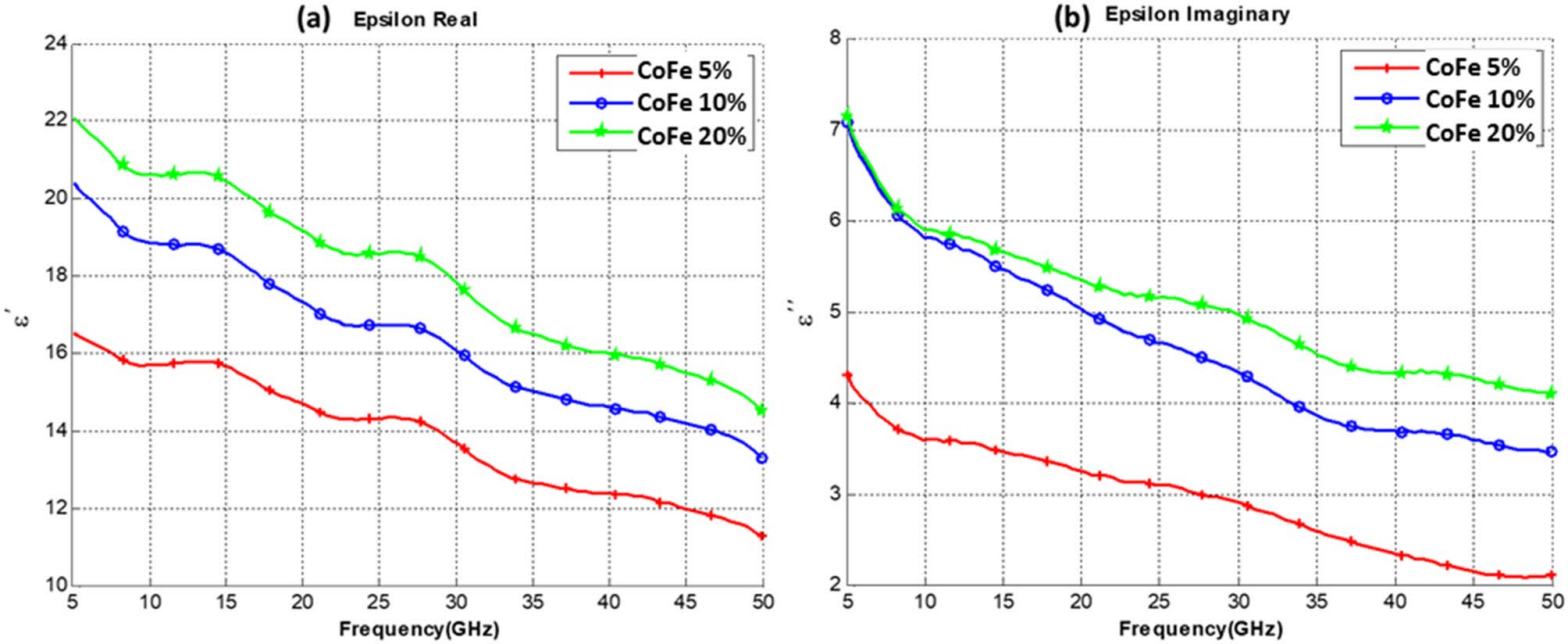
Dielectric Constant of PU and 5% CNT composites with different (CoFe) content. (**a**) Real Part. (**b**) Imaginary part.

Measurements were conducted on three sets of composite samples, all samples were made from 5 wt% of CNT mixed with PU, and three functionalizing elements were added to have 3 sets of samples. The functionalizing elements (Co, Fe, and CoFe) were added with 3 different concentrations of 5%, 10% and 20%. Thus each group of samples set consists of 3 different concentrations of the functionalizing element as illustrated in Table 1. The real part of the dielectric constant and the imaginary part of our CNT composites for sets 1, 2, and 3 are shown in Figs. 4, 5 and 6, respectively.

**Table 1:** Composite samples used in the measurement.

Set	Sample Specifications
Set 1	PU + 5% CNT with 5% Co oxide
	PU + 5% CNT with 10% Co oxide
	PU + 5% CNT with 20% Co oxide
Set 2	PU + 5% CNT with 5% Fe oxide
	PU + 5% CNT with 10% Fe oxide
	PU + 5% CNT with 20% Fe oxide
Set 3	PU + 5% CNT with 5% CoFe oxide
	PU + 5% CNT with 10% CoFe oxide
	PU + 5% CNT with 20% CoFe oxide

Observing the results obtained in Figs. 4, 5 and 6, one can notice that increasing the concentration of cobalt, an iron oxide or cobalt iron oxide as a functionalizing element will result in an increase in both the real and imaginary parts of the dielectric constant. It is also noted that iron oxide or cobalt iron oxide result in a higher dielectric constant than cobalt alone. Nonetheless, it is observed that the iron-oxide effect is closely similar to the CoFe effect. It is also noted that the curves exhibit a wavy shape, with several minima and maxima. Such a behavior has been previously reported for SWCNT/cross-linked polyurethane composites^14^. Complex permittivity properties of a material largely depend on contributions of various forms of polarizations such as interfacial, atomic, orientation and electronic. Also parameters such as percolation effect, CNT fiber aspect ratio, Nano-particle size, and other elemental parameters highly influence the complex permittivity properties.

### Reflection loss calculation

**Figure 7:**
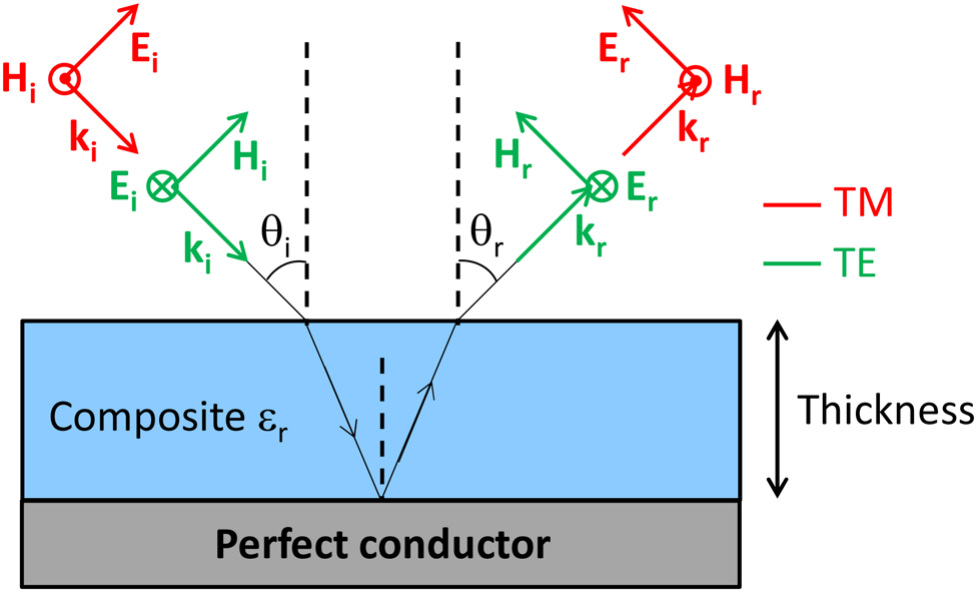
Reflection loss calculation: scheme of the reflection of an electromagnetic wave by a perfect conductor after penetrating a CNT composite layer.

The measured complex permittivity of the CNT composites will be used to estimate the absorption efficiency through the calculation of the reflection loss. As displayed in Fig. 7, we assume that an incident electromagnetic plane wave with two possible polarizations (TE and TM) penetrates a composite layer, characterized by CNT concentration and its thickness, and laid on top of a perfect conductor. The reflection loss calculation is done with the S matrix formalism^28^ will be adapted for treating the general case of multilayered media: the stack being represented by a 2 × 2 matrix whose coefficients are linked to the reflection and the transmission of the stack. From these coefficients, the reflectivity can be calculated, and thus the absorption efficiency of the composite layer can be derived. In the present work, only a single polyurethane/MWCNT composite layer is considered.

The reflection loss calculation confirms that the absorption spectrum can tuned by varying the CNT composite concentration or by changing the material thickness. Therefore, it is possible to strongly absorb the electromagnetic radiation in a spectral range of interest by a careful design of the composite layer, that is to say by choosing the suitable CNT composite concentration and thickness. Reflection loss calculations are conducted in the spectral range 5–50 GHz to estimate the absorption efficiency of our composites. The reflection loss calculation at normal incidence with different thickness (0.4–5 mm) of composite layer for the three sets of samples presented in Table 1. The effectiveness of absorption mechanism is the result of electromagnetic energy attenuation inside the sample. This is directly related to dielectric loss value, sample thickness, and impedance matching. Impedance matching is a concept used to maximize energy absorption by the load and minimize reflection. This occurs when the input impedance at the air and composite interface is equivalent to that of free space; ${Z}_{in}={Z}_{0}$, where

1 $$\frac{{Z}_{in}}{{Z}_{0}}=\sqrt{\frac{{\mu }_{r}}{{\varepsilon }_{r}}}tanh\left[j(\frac{\omega d}{c})\sqrt{{\mu }_{r}{\varepsilon }_{r}}\right]$$

It is clear that the input impedance ${Z}_{in}$ is dependent on the composite electric and magnetic properties, ${\varepsilon }_{r}$ and ${\mu }_{r}$, respectively , the sample thickness $(d)$ and the frequency $(\omega )$. The attenuation of the electromagnetic energy inside the sample is controlled by the attenuation factor ${e}^{-\alpha d}$, which works on attenuating (reducing) the wave amplitude with respect to traveling distance $d$. The attenuation constant $\left(\alpha \right)$ is also dependent on the electric and magnetic properties of the material and defined by

2 $$\alpha =\omega {\left\{\frac{\mu \varepsilon }{2}{\left[1+{\left(\frac{\sigma }{\omega \varepsilon }\right)}^{2}\right]}^{1/2}-1\right\}}^{1/2}$$

The reflection loss of EM radiation, under normal wave incidence at the surface of a single-layer material backed by a perfect conductor can be calculated as

3 $$RL\left(dB\right)=20 Log\left|\frac{{Z}_{in}-{Z}_{0}}{{Z}_{in}-{Z}_{0}}\right|$$

## Results and discussion

Using the dielectric constants obtained in Sec. III-A along with the reflection loss scenario illustrated in Sec. III-B one can obtain the effect of different composite concentration and composite layer thickness on the reflection loss behavior for the 3 sets of samples.

**Figure 8:**
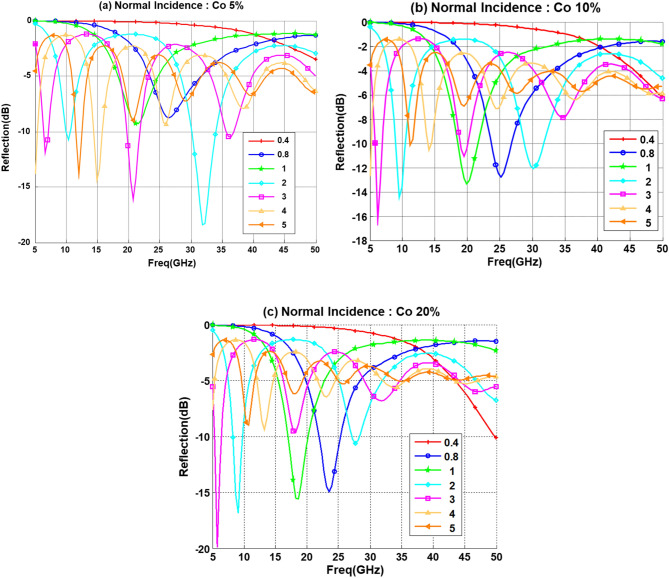
Reflection loss for PU with 5% CNT for different thickness (d mm) for (**a**) 5% Co. (**b**) 10% Co. (**c**) 20% Co.

Reflection loss calculation for samples with different concentration of functionalizing elements (Co) are presented in Fig. 8. It is observed that several minima of reflectivity appear in the considered spectral range (5–50 GHz) when the Co concentration inside the composite increases, and they are progressively shifted to the lower frequency band. This shift can be attributed to the increase in the dielectric constant value. This increase will result in a change in the input impedance of the composite layer, which will be translated into a shift in the reflection loss (RL) minima to lower frequency. The results display that an absorption level of − 10 dB can be attained in the 5–10 GHz range for samples with 10% and 20% Co concentrations with thickness between 2–3 mm. On the other hand composite layer thickness of 0.8 mm and 1 mm will have appreciable effect with 10% and 20% Co in the frequency range 18–24 GHz. The 5% Co appears to give good reflection loss at the 30–34 GHz range with a thickness of 2 mm.

**Figure 9:**
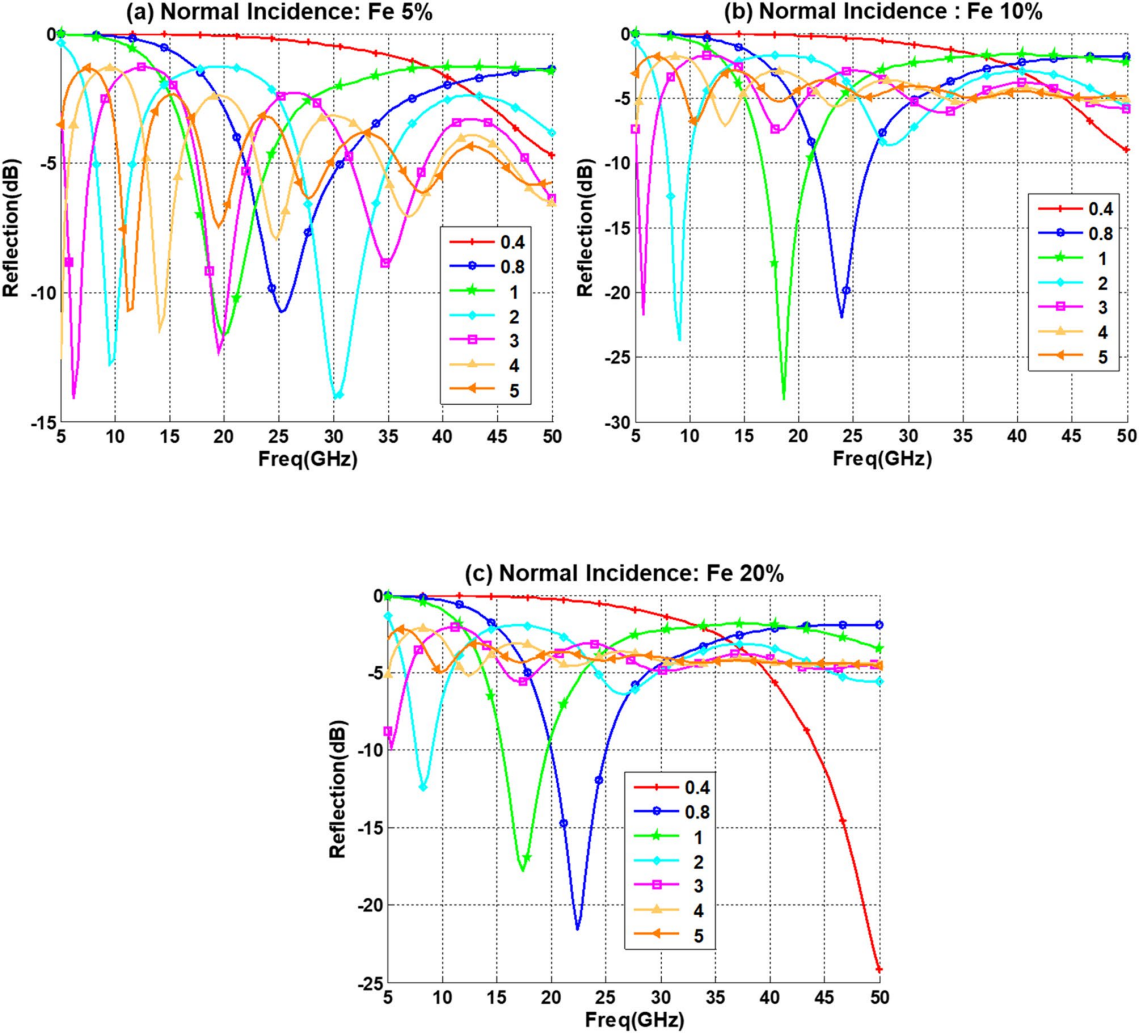
Reflection loss for PU with 5% CNT for different thickness (d mm) for (**a**) 5% Fe. (**b**) 10% Fe. (c) 20% Fe.

The effect of iron-oxide on the reflection loss for different composite thicknesses at normal incidence are given in Fig. 9. For Fe with 5% and 10% concentration we obtained a narrow band reflection loss between 5 and 10 GHz for 2 mm and 3 mm thickness, respectively, while at the same campsite thicknesses, these bands are not observed for 20% Fe. On the other hand, for a layer thickness of 0.8 mm the reflection loss is improving with increasing the Fe concentration from 5 to 20%. Similar to the Co behavior it is observed that the minima of reflectivity (maximum absorption) appear in the considered spectral range when the iron-oxide concentration increases, and they are progressively shifted to the lower frequency bands. However, it is noticeable that the number of minima is reduced by increasing the concentration from 5 to 20% as observed in Fig. 9b,c, this happens at smaller thickness such as 0.8 mm and 1 mm, and the smaller thickness effect (0.4 mm) on the reflection loss starts to appear at the upper band of the spectrum (50 GHz) with 20% iron-oxide concentration.

**Figure 10:**
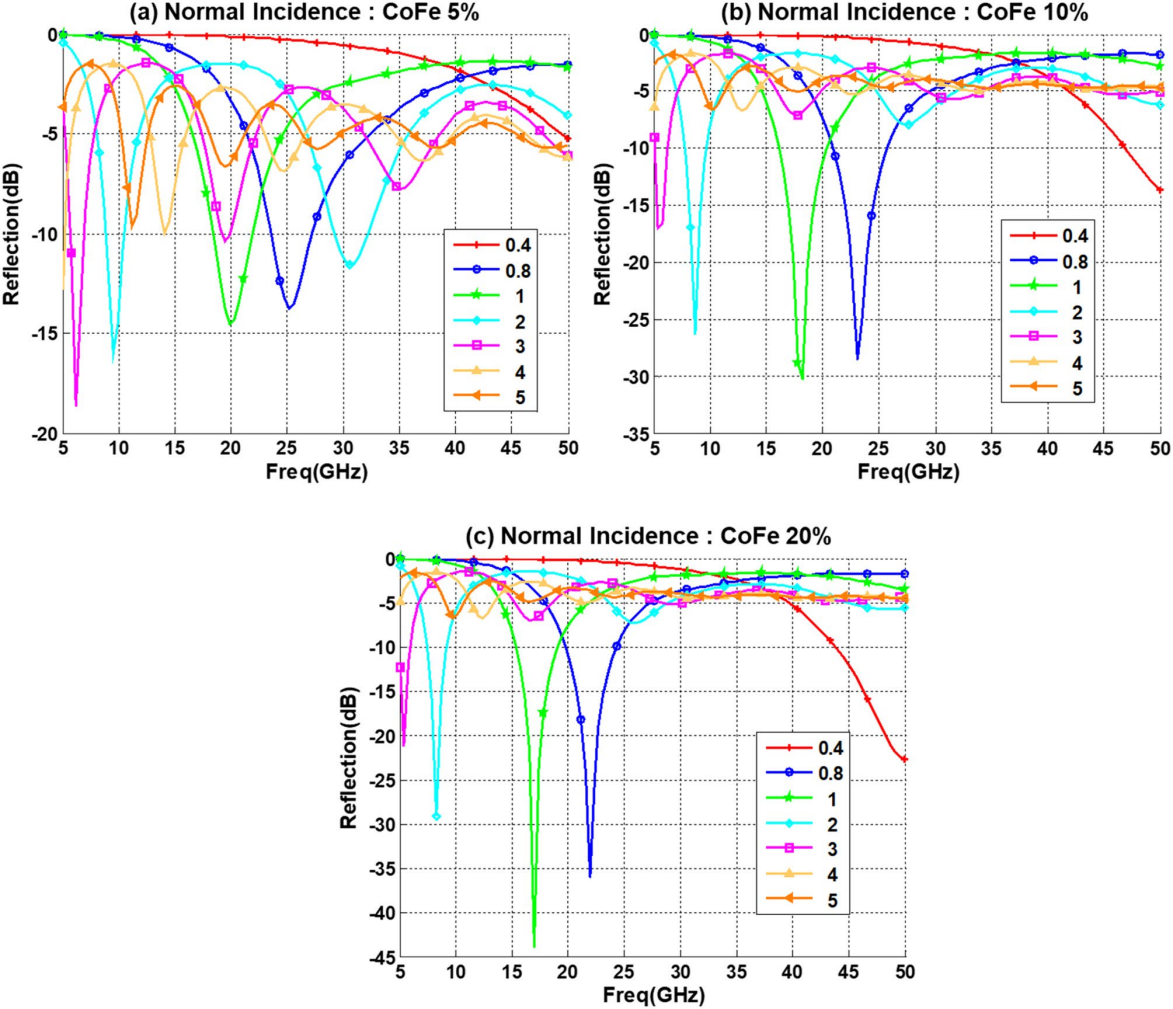
Reflection loss for PU with 5% CNT for different thickness (d mm) for (**a**) 5% CoFe. (**b**) 10% CoFe. (**c**) 20% CoFe.

The reflection loss behavior for CoFe composite with different concentration and layer thickness are given in Fig. 10. It can be concluded that CoFe exhibits the same behavior as the iron-oxide with increasing the concentration, but with higher sensitivity and narrower band. It is also evident that increasing the concentration will have negative effect on large thickness composite layers and positive effect on smaller thickness layer. This behavior is highly favorable for aeronautical applications, since this will results in a less weight composite.

## Conclusions

This paper describes the design procedure and development of microwave absorption material using functionalized CNT impeded in polyurethane polymer The CNT is functionalized with 3 types of metal alloyed nanoparticles (Co, Fe, and CoFe) with three different concentrations (5%, 10%, and 20%). The permittivity measurements of all 9 samples show that the developed composites have substantial dielectric losses when the functionalizing element content in the CNT increases. All 3 metal alloyed show improved reflection loss with 10% and 20% concentration. An absorption level of − 10 dB at normal incidence is reached by a composite layer with 3 mm at lower concentration and reduced to 0.8 mm with higher concentrations. Thus, functionalized CNT can be used to enhance and improve the absorption efficiency of the composite with the right composite thickness. It can lead to an extra gain of weight, which is a substantial breakthrough for aeronautics applications.
